# Association of the HDL lipidome with HDL traits before and after exercise training: HERITAGE family study

**DOI:** 10.1007/s11306-025-02330-3

**Published:** 2025-08-19

**Authors:** Prasun K. Dev, Eric C. Leszczynski, Charles S. Schwartz, Jacob L. Barber, Emanuel J. Ayala, Xuewen Wang, Ciaran M. Fairman, Sujoy Ghosh, Robert E. Gerszten, Michael Olivier, Anand Rohatgi, Clary B. Clish, Claude Bouchard, Mark A. Sarzynski

**Affiliations:** 1 Department of Exercise Science, University of South Carolina, Columbia, SC USA; 2 Division of Cardiovascular Medicine, Beth Israel Deaconess Medical Center, Boston, MA USA; 3 Pennington Biomedical Research Center, Baton Rouge, LA USA; 4 Broad Institute of Harvard and MIT, Cambridge, MA USA; 5 Center for Precision Medicine, Wake Forest School of Medicine, Winston-Salem, NC USA; 6 Department of Internal Medicine, University of Texas Southwestern Medical Center, Dallas, TX USA; 7 921 Assembly St, PHRC Rm 301B, Columbia, SC 29201, USA

**Keywords:** High-density lipoproteins, Lipids, Lipidomics, Sex differences

## Abstract

**Introduction:**

HDL particle functionality is influenced by its structure, including lipid composition. However, the effects of exercise training on the HDL lipidome and its relationship with HDL-related traits are largely unknown.

**Objective:**

To investigate the HDL lipidome of 154 adults before and after 20 weeks of endurance exercise training in the HERITAGE Family Study.

**Methods:**

The HDL-sized plasma fraction was isolated utilizing FPLC-SEC, followed by untargeted lipidomic analysis using LC/MS. A total of 11 HDL lipid classes were derived from the 341 identified known lipid species. Exercise response of the HDL lipidome and its associations with HDL-related traits were examined, with significance set to FDR < 0.05.

**Results:**

The abundance of 42 HDL lipid species at baseline and 43 at post-training were significantly different between males and females. Exercise training did not significantly alter the abundance of any HDL lipid class, although HDL phosphatidylethanolamine trended (FDR = 0.05) towards an increase. Two species of HDL diglycerides significantly decreased in the total sample. Sex-specific nominal (*p* < 0.05) changes in individual HDL lipid species included primarily HDL diglyceride and triglyceride species decreasing in males only, while HDL phosphatidylethanolamine species mostly increasing in females only. Higher abundance of HDL surface lipids was associated with larger size and cholesterol content of HDL particles before and in response to exercise training.

**Conclusion:**

Our analysis indicates that endurance exercise may have a limited impact on the HDL lipidome in healthy adults. However, the HDL lipidome differed across sex groups, which needs further investigation to identify potential mechanisms underlying the sex differences.

**Supplementary Information:**

The online version contains supplementary material available at 10.1007/s11306-025-02330-3.

## Introduction

Numerous epidemiological studies have found an inverse association between the concentration of high-density lipoprotein cholesterol (HDL-C) and cardiovascular disease (CVD) risk (Di Angelantonio et al., 2009; Toth et al., 2013; Wilson et al., 1988). Nonetheless, this association lacks a causal relationship as HDL-C raising therapies have failed to reduce CVD risk and Mendelian randomization studies have not found causal associations between HDL-C and CVD (Boden et al., 2011; Do et al., 2013; Tall & Rader, 2018; Toth et al., 2013; Voight et al., 2012). Consequently, HDL-focused research has shifted towards elucidating clinical biomarkers of the cardioprotective functions of HDL particles rather than the cholesterol content. HDL particles have many cardioprotective properties, such as anti-inflammatory, anti-oxidative, endothelial integrity, and the facilitation of reverse cholesterol transport (Bhatt & Rohatgi, 2016; Karlsson et al., 2015; Wu et al., 2022).

HDL are small, dense protein-rich particles made up of a phospholipid surface monolayer with a core comprised of cholesterol esters, triglycerides (TG), and free cholesterol. HDL particles carry a wide array of lipids, with profiling studies identifying over 200 lipid species collectively associated with HDL (i.e., HDL lipidome) (Kontush et al., 2013). Notably, the composition of HDL particles greatly influences their cardioprotective function. For instance, phospholipids and sphingomyelins have an affinity for cholesterol, thereby promoting the efflux of excess cholesterol from macrophages (Slotte, 1999; Yancey et al., 2000). Furthermore, previous studies have shown that the HDL lipidome is altered in several disease states, such as CVD, type 2 diabetes, and obesity (Lidgard et al., 2023; Mocciaro et al., 2022; Morgantini et al., 2014; Pruzanski et al., 2000). One study reported a low concentration of HDL phospholipids was associated with acute coronary syndrome and that whole plasma or lipoprotein lipidomic measures were superior to conventional risk factors in discriminating acute and stable coronary disease (Meikle et al., 2019). Collectively, these findings suggest that unraveling the intricate link between HDL lipid composition and HDL functionality is crucial for the development of targeted interventions aimed at treating and/or preventing CVD.

Regular endurance exercise represents a therapeutic intervention capable of reducing CVD risk in part by improving lipid and lipoprotein metabolism, including HDL metabolism (Adams et al., 2013; Blazek et al., 2013). Our group and others have reported improvements in HDL functionality in response to regular endurance exercise (Franczyk et al., 2023; Ruiz-Ramie et al., 2019; Woudberg et al., 2018). However, the extent to which exercise training alters the HDL lipidome and/or affects the relationship between the HDL lipidome and HDL function and other HDL properties remains mostly unknown. Previously, Khan et al. showed that a 12-week weight loss or aerobic exercise plus weight loss intervention in metabolic syndrome patients changed the HDL lipidomic profile closer to that of healthy individuals (Khan et al., 2018). However, this dietary intervention study in a smaller sample size study did not examine exercise alone, nor did it examine how changes in the HDL lipidome related to subsequent changes in HDL functionality. As such, in the current study, we aimed to investigate the association of the HDL lipidome with HDL function and other HDL related traits before and after a 20-week endurance exercise training intervention.

## Methods

### HERITAGE family study

A total of 855 physically inactive individuals from 218 family units who identified themselves as either Black or White were enrolled in the HERITAGE Family Study. These participants underwent a 20-week supervised endurance exercise training program at one of four clinical locations (Clinical trial registration #NCT00005137). The study design, inclusion and exclusion criteria, exercise training, and testing protocol has been previously described (Bouchard et al., 1995; Sarzynski et al., 2022). Participants were normotensive or mildly hypertensive (< 160/100 mm Hg) without medications for hypertension, diabetes, or dyslipidemia, and with a body mass index (BMI) below 40 kg/m^2^. The study protocol was approved by the Institutional Review Boards at each of the HERITAGE Family Study testing centers. Each participant signed an informed consent form, and all studies were carried out in line with the Declaration of Helsinki. A total of 742 participants from 204 family units were considered completers, as they finished the training program (completed at least 95% of the 60 required training sessions) and had complete or nearly complete data on all tests before and after training.

### Current study sample size

The current analysis utilizes specimens and data from 154 individuals out of the 742 completers as part of a funded ancillary project focused on HDL. These 154 participants (Female 61%, Black 29%; Table 1) were selected based on HDL training response (~ 50 participants each from the highest, middle, and lowest quartiles of the HDL response spectrum), cost-effectiveness, and availability of samples. Despite these criteria, this subsample still reflects the demographic composition of the original HERITAGE cohort, maintaining a balanced distribution by sex and race (Female 57%, Black 34%).

**Table 1:** Characteristics of HERITAGE participants in the total sample, males, and females at baseline and their changes following exercise training

	Total Sample (*n* = 154)			Males (*n* = 60)			Females (*n* = 94)			
	Baseline	Change	p-value	Baseline	Change	p-value	Baseline		Change	p-value
Race, % Black	29	–	–	21	–	–	35		–	–
Age, years	34 (13.1)	–	–	33.5 (14)	–	–	34.3 (12.6)		–	–
BMI, kg/m^2^	25.68 (4.8)	− 0.11 (0.8)	0.12	26.47 (4.9)	−0.03 (0.7)	0.72	25.18 (4.7)		−0.15 (0.9)	0.11
VO_2_max, mL/kg/min	32.58 (9.4)	37.88 (9.7)	4.2e^−55^	38.87 (9.5)	44.07 (10.0)	1.30e^−20^	28.51 (6.8)		33.83 (7.0)	5.2e^−36^
apoA-1, mg/dL	122.67 (16.8)	1.94 (13.2)	0.07	119.56 (15.2)	0.17 (13.1)	0.92	124.66 (17.5)		3.07 (13.2)	0.03
HDL-C, mg/dL	45.28 (13.2)	2.58 (7.4)	3.0e^−5^	40.57 (14.9)	1.12 (5.8)	0.14	48.29 (11.2)		3.52 (8.2)	6.93e^−5^
Triglyceride, mg/dL	100.36 (56.7)	− 3.81 (33.5)	0.16	114.97 (67.2)	− 6.41 (43.5)	0.26	91.03 (46.9)		− 2.14 (25.2)	0.41
Small HDL-P, nmol/L	12.28 (3.1)	− 0.48 (2.2)	0.01	13.37 (2.8)	− 0.53 (1.8)	0.03	11.59 (3.0)		− 0.44 (2.5)	0.08
Medium HDL-P, nmol/L	5.25 (2.1)	0.23 (1.8)	0.11	4.67 (2.3)	0.15 (1.7)	0.51	5.63 (1.9)		0.28 (1.8)	0.14
Large HDL-P, nmol/L	2.15 (1.3)	0.06 (0.7)	0.28	1.46 (1.1)	0.04 (0.6)	0.65	2.59 (1.3)		0.08 (0.8)	0.33
HDL Size, nm	9.04 (0.4)	0.02 (0.2)	0.32	8.80 (0.4)	0.01 (0.2)	0.58	9.19 (0.4)		0.02 (0.2)	0.41
Global Efflux	1.04 (0.3)	0.00 (0.2)	0.93	1.06 (0.2)	0.02 (0.2)	0.49	1.03 (0.3)		− 0.01 (0.2)	0.65
Non ABCA-1 Efflux	0.86 (0.2)	0.00 (0.2)	0.84	0.88 (0.2)	0.01 (0.2)	0.72	0.85 (0.2)		− 0.00 (0.1)	0.92
HDLox	0.03 (0.008)	− 0.002 (0.007)	0.01	0.03 (0.009)	1.1e^−5^ (6.0e^−4^)	0.99	0.03 (0.008)		− 0.003 (0.07)	0.001
Hepatic Lipase Activity, nmol/mL/min	187.85 (75.6)	− 8.46 (44.5)	0.02	238.83 (72.5)	− 16.24 (49.3)	0.02	154.99 (57.4)		− 3.31 (40.5)	0.45

*apoA-1* apolipoprotein A-1, *BMI* body mass index, *HDL-C* HDL cholesterol, *HDL-P* HDL particle concentration, *HDLox* HDL lipid peroxidation, *VO*_2_*max* maximal oxygen uptake. Baseline and change values presented as mean (SD). Change values represent the mean change in trait from baseline to post-training. p-values represent the significance of within-group changes in trait value following exercise training using paired t-tests

### Exercise testing protocol

All participants underwent two maximal exercise tests at each time point on an Ergo-Metrics 800 S cycle ergometer coupled to a SensorMedics 2900 metabolic cart (SensorMedics, Yorba Linda, CA, USA): (1) a maximal graded exercise test and (2) a combination submaximal/maximal test. Full details of the exercise testing protocols have been previously published (Sarzynski et al., 2022). The average maximal oxygen uptake (VO_2_max) from the two maximal exercise tests was used as the VO_2_max for that subject and used in analyses if both values were within 5% of each other. If they differed by > 5%, the higher VO_2_max value was used.

### Exercise training program

The exercise training program consisted of three weekly sessions on a stationary bicycle ergometer (Universal Aerobicycles, Cedar Rapids, IA) at a heart rate corresponding to 55% of baseline VO_2_max for a 30-minute duration for the first two weeks. Exercise session duration and intensity were progressively increased every two weeks until a heart rate corresponding to 75% of baseline VO_2_max for a 50-minute duration was attained. This workload was maintained for the final six weeks of training. Power output was controlled directly relative to heart rate by using the Universal Gym Mednet (Cedar Rapids, IA) computerized system. The protocol was standardized across all participating centers and properly supervised to ensure that the equipment was working properly.

Blood samples were collected following a 12-hour fast at baseline and at least 24 h after completing the last exercise session.

### Isolation of HDL-sized plasma fraction

The HDL-sized plasma fraction was isolated from whole plasma using fast protein liquid chromatography (FPLC) with size exclusion chromatography as previously described (Gordon et al., 2010). Briefly, ~ 370 µL of whole plasma was injected into an Akta Pure FPLC (GE Healthcare) system with three gel filtration columns (Superdex 200 increase columns, GE Healthcare) arranged in series. The HDL-sized plasma fraction was collected and concentrated using 10 kDa molecular weight cut off centrifugal filters (Amicon, Millipore Sigma) as previously described (Michell et al., 2016). The concentrated HDL-sized sample (hereby referred to as HDL) was then used for mass spectrometry analysis.

### HDL lipidome profiling

Detailed methods for the measurement of the lipidome have been previously described (Wang et al., 2018). Briefly, positive ion mode (C8-pos) analyses of polar and non-polar plasma lipids were conducted by the Broad Institute Metabolic Platform using a LC-MS system composed of a Shimadzu Nexera X2 U-HPLC (Shimadzu Corp) coupled to an Exactive Plus orbitrap mass spectrometer (Thermo Fisher Scientific). Lipids were extracted from HDL plasma (10 µL) using isopropanol containing 1,2-didodecanoyl-sn-glycero-3-phosphocholine as an internal standard (Avanti Polar Lipids) and after centrifugation supernatants were injected directly onto a ACQUITY BEH C8 column (Waters). MS analyses were performed using electrospray ionization in the positive ion mode using full scan analysis over 200 to 1000 m/z at 70,000 resolution and 3 Hz data acquisition rate.

To allow for quality control analyses (QC) and drift correction, pairs of pooled plasma QC samples were included every 20 samples and results were standardized using the ratio of the value of the sample to the value of the nearest pooled reference multiplied by the median of all reference values for the lipid metabolite. One pooled QC sample from each pair was used to correct for analytical drift using a nearest neighbor approach and the second pooled plasma sample served as a passive QC sample to evaluate analytical reproducibility of every lipid. Raw data from Exactive Plus instruments were processed using TraceFinder software (Thermo Fisher Scientific) and Progenesis QI (Nonlinear Dynamics). Since reference standards were not available to confirm identities of all measured lipid species, representative lipids from each lipid class were used to characterize retention time (RT) and mass to charge ratio (m/z) ratio patterns. Lipid identities are reported at the level of lipid class, total acyl carbon content, and total double bond content since the LC-MS method does not discretely resolve all isomeric lipids from one another.

Only named lipid species were considered for statistical analysis. A total of 341 named lipid species were identified, and their names and m/z and RT values can be found in Supplementary Table S1.

#### HDL lipid class

A total of 11 major HDL lipid classes were identified, comprising 335 HDL lipid species. An additional 6 HDL lipid species were unclassified and not included in this categorization. HDL lipid species identified were: LPC: lysophosphatidylcholine (32 species), LPE: lysophosphatidylethanolamine (3), PC: phosphatidylcholine (102), PE: phosphatidylethanolamine (28), Cer: ceramide (5), HexCer: hexosylceramide (4), SM: sphingomyelin (34), CE: cholesterol ester (25), MG: monoacylglycerol (8), DG: diglyceride (27), and TG: triglyceride (67). HDL surface fluidity was defined as SM to PC ratio (Zhu et al., 2019). The abundance of each lipid class is the summation of the abundance of each lipid species within the same lipid class. Furthermore, the calculated abundance of each lipid species and derived lipid class was normalized to the corresponding plasma apoA-I values of each participant as previously described (Chapman et al., 2024).

### HDL-related trait measurements

#### Lipids, lipoproteins, and lipase activity

As previously described (Pérusse et al., 1997), total cholesterol and TG levels were determined in plasma and lipoprotein fractions by enzymatic methods using the Technicon RA-1000 analyzer. Concentrations of apoA-I in plasma and lipoprotein fractions were measured by the rocket-immunoelectrophoretic method. Post-heparin hepatic lipase (HL) activity was measured separately from the blood drawn for lipid measures. Activity was measured after a 12-hour overnight fast and 10 min after an intravenous injection of heparin (60 IU/kg body mass) as previously described (Pérusse et al., 1997).

#### HDL subclass profiling

The HDL subclass profile was quantified via NMR spectroscopy at LabCorp, Inc (Morrisville, N.C.) using the LP4 deconvolution algorithm (Jeyarajah et al., 2006).

#### Cholesterol efflux capacity assay

Measurement of the efflux of radiolabeled (^3^H) cholesterol from J774 macrophages to apoB depleted plasma in the presence and absence of cAMP was performed as previously described (Khera et al., 2011).

#### HDL oxidation

Lipid peroxidation (HDLox), which represents the HDL lipid peroxide content, was measured in apoB depleted serum using the Amplex^®^ Red cholesterol assay (Invitrogen) following previously described methods (Kelesidis et al., 2014). Higher HDLox values are associated with reduced HDL antioxidant function.

### Statistical analysis

All analyses were performed both on HDL lipid classes and individual HDL lipid species, however lipid classes were the primary focus of the analysis. HDL lipid class and species abundance data were log_2_ transformed., but log_2_ transformed HDL lipid data were still mostly non-normally distributed, thus non-parametric models were used. Wilcoxon signed-rank tests were used to examine changes in the HDL lipidome in response to exercise training, while paired t-tests were used for clinical HDL traits. Spearman correlations were used to test the associations of HDL lipid classes with HDL related traits. All correlation analyses were performed both at baseline and in response to exercise training (i.e., change in HDL lipid with change in HDL trait), with adjustments made for age, sex, and race. Analyses for change also included baseline HDL lipid value and baseline trait value as covariates to represent the true association of change in HDL lipid with change in HDL phenotype independent of their baseline values. Given known differences in HDL profiles among males and females, all analyses were performed in the total sample and stratified by sex, with sex-stratified models no longer adjusting for sex. Moreover, differences in HDL lipid classes and species between sex groups at baseline and post-training were examined using Wilcoxon rank-sum tests. The significance threshold was set to false discovery rate (FDR) < 0.05 using the Benjamini-Hochberg procedure for all analyses. Statistical analyses were carried out using SAS 9.4 and R 4.4.1 statistical software.

## Results

The descriptive and clinical characteristics of the 154 participants before and after 20 weeks of endurance exercise training can be found in Table 1. In general, exercise training significantly altered the HDL profile in this subsample of HERITAGE, including increases in HDL-C and decreases in small HDL particles, HL activity, and HDLox (Table 1).

### HDL lipid classes at baseline and changes with training

HDL-PC was the most abundant and HDL-LPE the least abundant HDL lipid class at both baseline and post-training. No HDL lipid class significantly changed with exercise training in the total sample (Table 2), though the increase in HDL-PE was marginally significant (FDR adjusted *p* = 0.05, *p* = 0.005) and HDL-DG nominally trended (*p* < 0.05) towards a decrease (FDR-adj *p* = 0.21, *p* = 0.04). At both baseline and post-training, female participants had significantly higher HDL-SM levels compared to male participants, while nominal (*p* < 0.05) sex differences were observed for HDL-HexCer at both time points (Table 2). At baseline, nominal (*p* < 0.05) sex differences in HDL lipid class abundance were observed for HDL- Cer, DG, and LPE, while HDL- PC and PE showed nominal sex differences at post-training (Table 2). Within sex groups, HDL-PE trended (FDR-adj *p* = 0.06) towards an increase with training in females only (Table 2).

**Table 2:** Mean abundance of HDL lipid class at baseline and post training in the total sample and within sex groups

HDL Lipid Class	Baseline				Post-training			
	Total	Males	Females	*p*-value/FDR	Total	Males	Females	*p*-value/FDR
LPC	23.04 (0.5)	23.08 (0.5)	23.01 (0.5)	0.40/0.55	23.04 (0.5)	23.11 (0.6)	23.00 (0.4)	0.21/0.29
LPE	13.39 (1.2)	13.27 (1.2)	13.47 (1.3)	0.048/0.09	13.44 (1.4)	13.10 (1.8)	13.66 (0.9)	0.09/0.16
PC	30.14 (0.7)	30.06 (0.6)	30.19 (0.7)	0.10/0.16	30.15 (0.8)	29.96 (1.0)	30.27 (0.5)	0.04/0.11
PE	21.75 (0.8)	21.67 (0.7)	21.80 (0.8)	0.051/0.09	21.86 (0.9)*	21.63 (1.1)	22.00 (0.6)^#^	0.02/0.1
Cer	17.07 (0.9)	16.94 (0.7)	17.14 (0.9)	0.02/0.09	17.10 (0.9)	16.88 (1.2)	17.25 (0.7)	0.051/0.11
HexCer	17.84 (0.8)	17.72 (0.8)	17.91 (0.8)	0.03/0.09	17.87 (0.9)	17.65 (1.2)	18.01 (0.7)	0.03/0.1
SM	25.14 (0.8)	24.95 (0.8)	25.25 (0.9)	0.002/0.03	25.19 (0.9)	24.91 (1.1)	25.37 (0.7)	0.003/0.03
CE	25.58 (0.3)	25.59 (0.2)	25.57 (0.3)	0.80/0.80	25.55 (0.3)	25.54 (0.4)	25.57 (0.3)	0.20/0.29
MG	20.80 (0.7)	20.77 (0.8)	20.81 (0.7)	0.73/0.8	20.79 (0.8)	20.70 (1.0)	20.85 (0.7)	0.43/0.48
DG	21.20 (0.6)	21.31 (0.6)	21.12 (0.6)	0.04/0.09	21.09 (0.7)^#^	21.07 (0.8)	21.10 (0.6)	0.54/0.54
TG	24.99 (0.6)	25.02 (0.6)	24.98 (0.6)	0.45/0.55	24.93 (0.7)	24.85 (0.8)	24.98 (0.6)	0.41/0.48

All values presented as mean (SD) of log_2_ data. Post-training refers to outcomes measured after the 20 weeks of exercise training. p-value/FDR represent the mean difference in HDL lipid class abundance between males and females via Wilcoxon rank-sum test at each timepoint. FDR represents the p-value adjusted for multiple testing using the Benjamini Hochberg method, while p-value represents the unadjusted value. *Represents significant (FDR < 0.05) and ^#^represents nominally significant (*p* < 0.05) change in HDL lipid class in response to exercise training calculated via Wilcoxon signed-rank test between paired post-training and baseline measures. *CE* cholesterol ester, *Cer* ceramide, *DG* diglyceride, *HexCer* hexosylceramide, *LPC* lysophosphatidylcholine, *LPE* lysophosphatidylethanolamine, *MG* monoacylglycerol, *PC* phosphatidylcholine, *PE* phosphatidylethanolamine, *SM* sphingomyelin, *TG* triglyceride

### Significant associations of HDL lipid classes with HDL traits

**Fig. 1:**
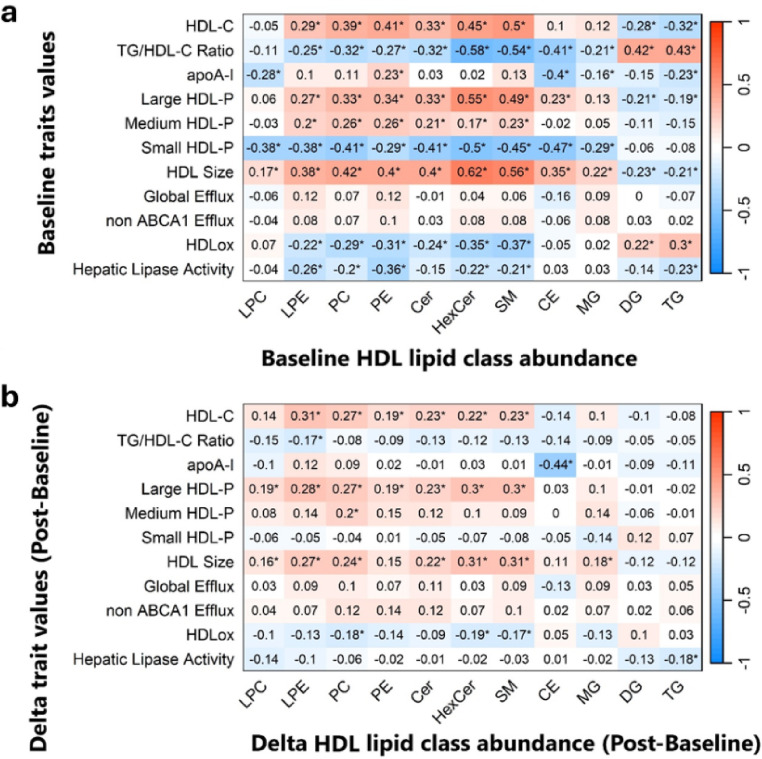
Spearman correlation values between HDL lipid classes (x-axis) and HDL traits (y-axis). **a** Correlation between baseline HDL lipid classes and baseline HDL-related traits while adjusting for age sex, and race. **b** Correlation between exercise training induced change in abundance of HDL lipid classes and change in HDL-related traits while adjusting for age sex, race, and baseline HDL-related traits and baseline HDL lipid class. *Represents FDR<0.05. Analysis utilized log_2_ transformed values of lipid class abundance normalized by the corresponding plasma apoA-I. *LPC* lysophosphatidylcholine, *LPE* lysophosphatidylethanolamine, *PC* phosphatidylcholine, *PE* phosphatidylethanolamine, *Cer* ceramide, SM: sphingomyelin, *CE* cholesterol ester, *MG* monoacylglycerol, *DG* diglyceride, *TG* triglyceride, *HDL-C* HDL cholesterol, *HDL-P* HDL particle, *HDLo*: HDL lipid peroxidation

HDL- LPE, PC, PE, Cer, HexCer, and SM were associated (*ρ* = 0.29 to 0.62, FDR < 0.05) with the concentration of HDL-C and large HDL particles and mean HDL size at baseline (Fig. 1A). Similarly, in response to exercise training, changes in HDL- LPC, LPE, PC, Cer, and SM were associated with concomitant changes in these same three HDL traits (*ρ* = 0.16 to 0.31, FDR < 0.05; Fig. 1B). Notably, HDL- LPE, PC, PE, HexCer, and SM were inversely associated with HDLox and HL activity (*ρ*= −0.20 to −0.37, FDR < 0.05) at baseline (Fig. 1A) and changes in these HDL lipid classes were also inversely associated with change in HDLox in response to exercise training (*ρ*= −0.17 to −0.19, FDR < 0.05; Fig. 1B). None of the HDL lipid classes were associated with cholesterol efflux capacity. Moreover, the HDL SM to PC ratio was positively associated with HDL-C and large HDL particle concentration and HDL size (*ρ* = 0.52 to 0.63, FDR < 0.05), and negatively associated with TG/HDL-C ratio, small HDL particles, HDLox, and HL activity (*ρ*=−0.19 to −0.72, FDR < 0.05) at baseline (Supplementary Table S2).

### Sex-specific significant associations of HDL lipid classes with HDL traits

At baseline, males and females showed similar associations, both in terms of strength and direction, between most HDL lipid classes and HDL traits. For example, HDL PC, PE, CER, HexCer, and SM were positively associated with HDL-C and mean HDL size in males (*ρ* = 0.19 to 0.64, FDR < 0.05) and females (ρ = 0.34 to 0.62, FDR < 0.05) (Supplementary Figure S1). However, at baseline HDL-TG was inversely correlated with both large and medium HDL concentration in males (*ρ*= −0.32 and − 0.35, respectively, FDR < 0.05), but not associated in females. Similarly, HDL-DG and HDL-TG were positively correlated with HDLox at baseline in males, but not females. For exercise response associations, fewer HDL lipid classes were associated with HDL traits in females compared to males (Supplementary Figure S1). Exercise-induced change in HDL-DG was positively associated with change in small HDL particle concentration in females (*ρ* = 0.29, FDR < 0.05) only. Changes in HDL surface lipids (e.g., PC, PE, SM, Cer, LPC) were negatively associated with concomitant change in HDLox in males (*ρ*= −0.18 to −0.30, FDR < 0.05), while in females only HDL-PC, PE, and HexCer were negatively associated with change in HDLox (*ρ*=−0.16 to −0.19, FDR < 0.05). Furthermore, changes in medium HDL concentration and TG/HDL-C were associated with concomitant changes in most HDL lipid classes in males, with only two HDL lipid classes associated in females. Conversely, change in large HDL particle concentration was associated with changes in almost all HDL surface lipids in females (*ρ =* 0.25 to 0.38, FDR < 0.05), but only associated with changes in HDL-LPC and HDL-LPE in males (*ρ* = 0.25 and 0.31, FDR < 0.05) respectively. The full results of our sex-stratified analyses can be found in Supplementary Figure S1.

### HDL lipid species changes with training

**Fig. 2:**
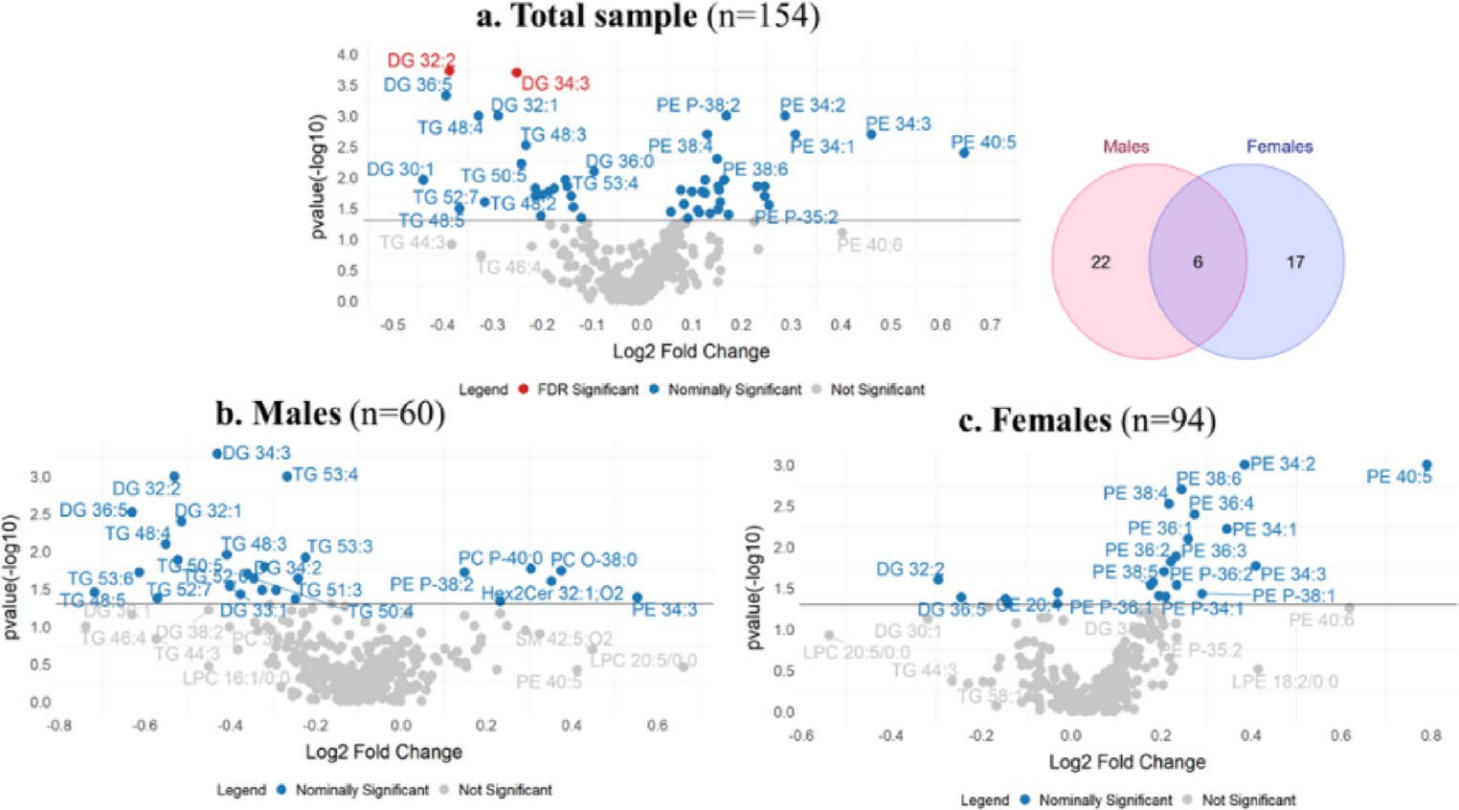
Magnitude (x-axis) and significance (y-axis) of change in HDL lipid species in the **a** total sample and **b** male and **c** female participants in response to exercise training. Only DG 32: and DG 34:3 significantly changed (FDR<0.05) in the total sample, with all other changes being nominally significant (*p*<0.05, indicated by horizontal line). The Venn diagram in the top right shows the overlap of the number of HDL lipid species that nominally changed across males and females. p-value represents whether the change with training was significant based on Wilcoxon signed-rank tests in the total sample and within sex groups. Analysis utilized log2 transformed values of lipid species abundance normalized by the corresponding plasma apoA-I

Two HDL-DG species significantly changed with exercise training in the total sample, while another 48 species nominally changed, mostly species from the PE (20), TG (11), DG (10), and PC (5) classes (Fig. 2A, Supplementary Table S3).

### Sex differences in HDL lipid species

**Fig. 3:**
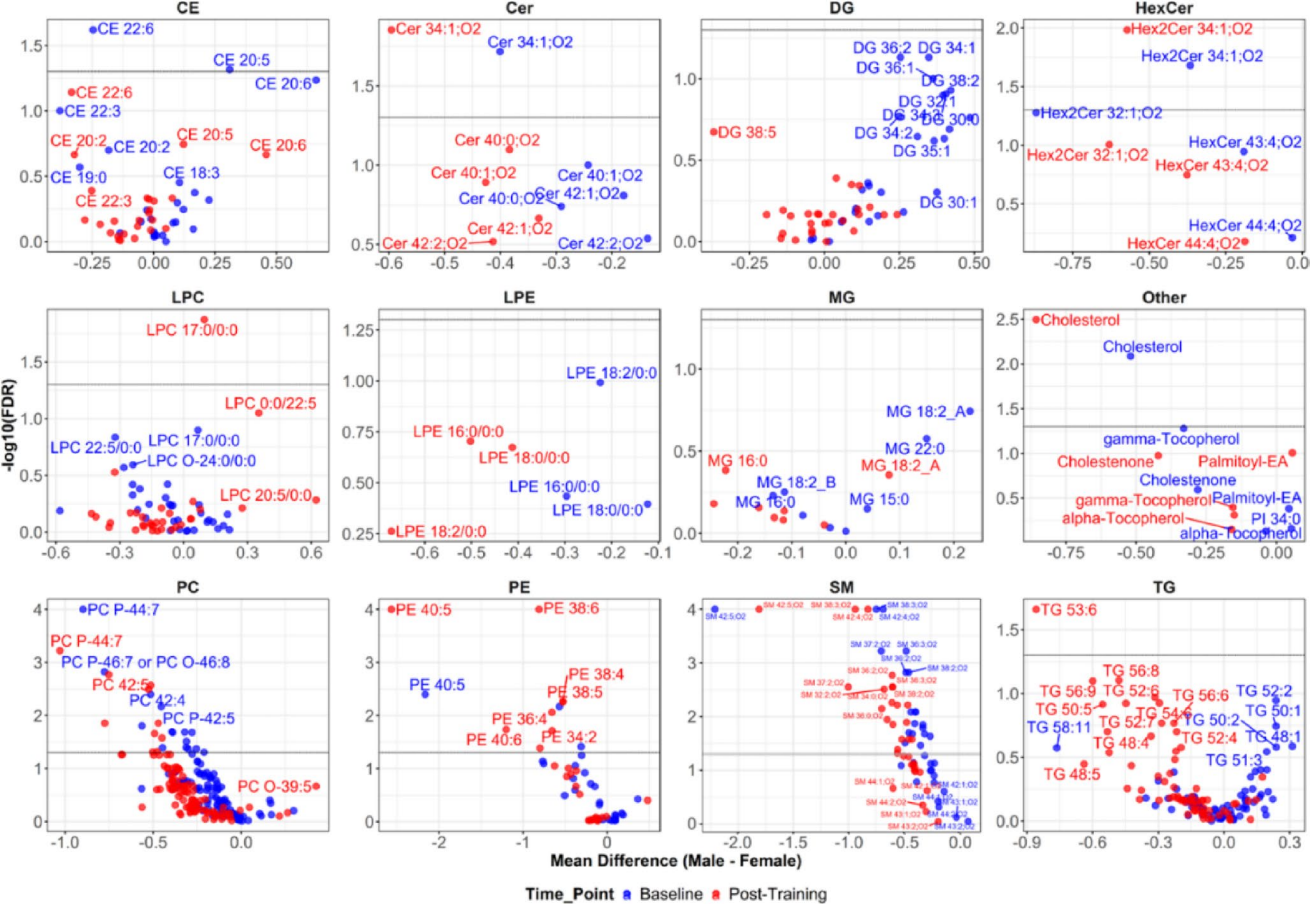
Differences in HDL lipid species abundance within each lipid class between males and females at baseline and after 20 weeks of exercise training. The x-axis represents the mean difference in abundance between males and females for each species. Points on the negative side indicate higher abundance in females, while those on the positive side indicate higher abundance in males. Significance (indicated by horizontal line; FDR p-values, y-axis) of difference in abundance was obtained using Wilcoxon rank-sum test of baseline and post-training values of lipid species. Analysis utilized log_2_ transformed values of lipid species abundance normalized by the corresponding plasma apoA-I. *CE* cholesterol ester, *Cer* ceramide, *DG* diglyceride, *HexCer* hexosylceramide, *LPC* lysophosphatidylcholine, *LPE* lysophosphatidylethanolamine, *MG* monoacylglycerol, *PC* phosphatidylcholine, *PE* phosphatidylethanolamine, *SM* sphingomyelin, *TG* triglyceride

We observed statistically significant (FDR < 0.05) sex differences in the abundance of 42 HDL lipid species at baseline and 43 HDL lipid species at post-training, with 30 lipids different between sex groups at both time points (Fig. 3, Supplementary Table S4). Sex-stratified analysis revealed that no individual HDL lipid species significantly changed with training in male or female participants. However, 13 HDL TGs, 9 DGs, 3 PCs, 2 PEs, and one HexCer nominally changed in males, whereas 17 HDL PEs, 4 DGs, and one CE and SM each nominally changed in females in response to exercise training (Fig. 2B, C, Supplementary Table S3).

To better understand the observed differences in HDL lipids between males and females, we conducted general linear models (GLM) to examine whether the 42 HDL lipid species would still exhibit sex differences in abundance after being adjusted for clinical measures with observed sex differences in HERITAGE. Specifically, we found that measures of body composition (i.e., total abdominal fat area, percent body fat, waist-to-hip ratio), sex steroid hormones (i.e., dehydroepiandrosterone, dihydrotestosterone, progesterone, and testosterone), and HDL subclass particle concentrations (i.e., small, medium, and large HDL particle concentrations) differed between HERITAGE female and male participants (Supplementary Table S5). We performed three GLM models for each lipid species to evaluate the association between these HDL lipid species and the aforementioned clinical measures groups as covariates (one model for body composition covariates, one for hormones, one for HDL subclasses). Each model also included sex, race, and age as common covariates, with the goal to examine whether sex remained significantly associated with the HDL lipid after adjustment for potential confounding. In models that separately adjusted for body composition or sex hormone measures, sex remained independently associated with almost all of the 42 HDL lipid species with the exception of 3 species in body composition models and 5 species for hormone models (Supplementary Table S6). Conversely, when HDL subclass concentrations were included as covariates, the sex difference only remained for 5 HDL lipid species, indicating the potential contribution of sex differences in HDL particle size distribution to the observed sex differences in these HDL lipids (Supplementary Table S6). Results were similar when applied to all 11 HDL lipid classes (Supplementary Table S7).

## Discussion

Prior research has shown that both HDL lipid composition and functionality are associated with cardiometabolic health (Lidgard et al., 2023; Mocciaro et al., 2022; Rohatgi et al., 2021; Yetukuri et al., 2010). However, there is limited evidence of how regular exercise affects the HDL lipidome and whether exercise-induced changes in the HDL lipidome are associated with concomitant changes in HDL functionality. In this study, we provide a detailed characterization of HDL lipids and related HDL traits, including two measures of HDL function, before and after 20 weeks of regular exercise. Our findings recapitulate associations between HDL lipid classes and HDL-related traits and importantly illustrate potential roles for changes in select HDL lipid classes in exercise-induced changes in HDL function, metabolism, and subclass distribution. We also found evidence of novel sex differences in the HDL lipid species that persisted even after exercise training.

Our findings revealed that a higher abundance of HDL surface lipids (i.e., PC, PE, SM, and Cer) was associated with larger size and cholesterol content of HDL before and in response to exercise training. Previous studies have shown that the fluidity of HDL is crucial for its ability to transfer phospholipid hydroperoxides from LDL to HDL, where the SM to PC ratio serves as a proxy for the fluidity of HDL particle (Kontush et al., 2013). Interestingly, we found that the SM to PC ratio was negatively associated with HDL lipid peroxidation at baseline. These findings are in line with previous studies (Kontush et al., 2013) that emphasize the functional role of HDL surface lipids in the various cardioprotective functions of HDL particles (e.g., anti-oxidant and anti-inflammatory activities).

Endurance exercise training did not significantly alter the abundance of any HDL lipid classes or individual HDL lipid species, though there were nominal changes in individual lipid species in the total sample. One previous study by Khan et al. found that a combined diet and aerobic exercise intervention in subjects with metabolic syndrome significantly changed the HDL HexCer class, while changes at the individual lipid species level were primarily observed in PCs and TGs (Khan et al., 2018). However, in contrast, we found that mostly HDL PEs, DGs, and TGs nominally changed in response to exercise training.

Overall, our findings suggest that endurance exercise training alone may have a limited impact on the HDL lipidome, whereas combining diet with exercise training could be a more effective strategy for achieving significant and targeted changes in the HDL lipidome. Previous work from our group and others have shown exercise volume is important for increasing HDL-C levels while exercise intensity may be more important for improving cholesterol efflux capacity (Ruiz-Ramie et al., 2019). The current study only examined one dose of exercise (same number of days per week at the same relative intensity level) and did not strictly control diet. Thus, it is possible that the amount and/or intensity of the exercise program in HERITAGE was not sufficient to induce substantial alterations in the HDL lipidome. The intensity of exercise in HERITAGE was vigorous for the last six weeks and this subset of HERITAGE participants did experience large increases in VO_2_max and HDL-C, showing the exercise intervention was indeed successful. However, this subset did not significantly decrease plasma TG levels, which may be an indication that lipids (plasma or HDL) were not largely impacted in this group. Although it is difficult to control diet in exercise intervention studies, dietary patterns were measured at baseline and post-training in HERITAGE and participants were instructed at the beginning and mid-training to not change their baseline health habits and continue their usual eating patterns. It was previously reported in HERITAGE that dietary lipid consumption was relatively low and no significant change in dietary lipid uptake was observed on repeated assessments (Leon et al., 2000). We also observed that body weight did not change in this subsample. In general, these HERITAGE participants would be considered healthy, as they were free of overt disease and had lipid profiles in the normal range. Thus, another possibility is that their HDL lipidome profile was already within an ‘optimal’ range, with less room for improvement than individuals with cardiometabolic impairments or disease.

The HDL lipidome is a relatively novel area of study, and subsequently little is known about the changes in HDL composition and subsequent CVD risk. Previous studies of the HDL lipidome and health outcomes have documented significant associations with cardiometabolic measures. For example, one study by Mocciaro et al. found that patients with central obesity and metabolic syndrome had higher levels of HDL DGs, TGs, and PCs (Mocciaro et al., 2022). Given the well-known benefits of exercise training, it is not surprising we observed nominal reductions in multiple HDL-DGs and TGs, which likely contributed to the overall reduction in plasma TG observed in the HERITAGE study.

We found stronger evidence of sex differences at the individual HDL lipid species level than the HDL lipid class level, which only showed nominal differences. For example, there were nominal differences in the abundance of HDL-SM at both baseline and post-training timepoints, however, at the species level, several individual HDL-SMs showed significant differences in abundance between males and females. Notably, the observed sex differences in HDL lipid species were independent of training status, as well as body composition and sex steroid hormones. However, sex differences in HDL lipid species were attenuated when adjusting for HDL subclass concentrations. These results suggest that inherent sex-based physiological differences do not seem to contribute to the observed sex differences in HDL lipids, but sex differences in HDL particle size distribution may underlie our sex-specific findings. Several previous studies have reported sex-specific differences in circulating lipids (Barranco-Altirriba et al., 2024; Slade et al., 2021; Tabassum et al., 2023). Of these, the study by Slade et al. reported higher plasma levels of PC, PE, PI, PG, SM, GlcCer, and CE in females, while higher TG and DG were observed in males (Slade et al., 2021). Moreover, regarding the HDL lipidome, only one study that conducted a four-week omega-3 dietary intervention reported sex differences. At baseline, the authors found a higher abundance of HDL lipid classes PC, PE, SM, and Cer in females compared to males, and a higher abundance of HDL DG and TG in males compared to females (Padro et al., 2023). Similarly, in our study, a nominally higher abundance of HDL PC, PE, SM, and Cer was observed in females compared to males after exercise training.

A strength of this study is the sample size (*n* = 154; 94 females, 60 males), as previous studies examining the HDL lipidome and lifestyle interventions had fewer participants (e.g., 53 with metabolic syndrome (Khan et al., 2018). Additionally, all participants were fully supervised during the exercise training intervention with high adherence. This study is the first to highlight potential sex differences in the HDL lipidome before and in response to endurance exercise training. However, our study also has some limitations, as the participants were relatively healthy, and the study did not include a control group. As such, the results of this study cannot be applied to the general population and thus further investigation in a larger and more diverse population is warranted. For cost-effectiveness, we only utilized positive ion mode for lipidomic analysis, which does not detect anionic lipids such as free fatty acids, phosphatidylserines, and phosphatidylinositols. Thus, our coverage of the HDL lipidome was not complete and the missing lipid classes could respond differently to regular exercise compared to those that were measured. Although we used rigorous quality control measures and reference standards, the full scan method is unable to unambiguously characterize the fatty acid constituents of isomeric species (e.g., TGs, DGs, PCs, ceramides). Since we did not use MS/MS validation of lipid species, this could lead to misannotation, particularly for isomeric lipids. In contrast, the molecular weight of each LPC, LPE, CE, and SM analyte identifies a specific acyl chain length and saturation (Rhee et al., 2011). Another limitation is that we isolated HDL based on size and not density, and thus this fraction may not consist exclusively of HDL associated lipids. Moreover, since our method standardized volume of plasma injected not HDL mass, we normalized our HDL lipid abundance data to plasma apoA-I levels to at least partially account for differences in HDL mass/particle number across individuals. However, this may limit our ability to determine whether the observed HDL lipid changes are truly due to compositional changes vs. simply changes in the concentration of lipids per particle and/or number of particles.

In conclusion, our comprehensive analysis indicates that moderate-to-vigorous endurance exercise training appears to have a limited impact on the HDL lipidome in healthy adults. Importantly, our analysis revealed several associations between the HDL lipidome and HDL traits that were observed at both baseline and in response to exercise training, indicating the stability of these relationships even after regular exercise. Additionally, we found evidence suggesting the abundance of HDL lipids differed across biological sex groups, particularly at the species level. Thus, our findings suggest a more granular examination of the HDL lipidome may reveal physiological differences not observed when quantifying lipids at the class level. Further investigation of potential sex differences in the HDL lipidome and the underlying mechanisms of these differences are warranted to confirm our preliminary findings. Moreover, future studies in populations across the cardiometabolic health spectrum are needed to determine whether the HDL lipidome may need a different exercise stimulus, combined exercise and diet stimulus, and/or the co-occurrence of weight loss to be altered substantially.

## Supplementary Information

Below is the link to the electronic supplementary material.

Supplementary Material 1

## Data Availability

The datasets used for the analysis in the current study are available from the corresponding author on reasonable request.
